# Spatial Vertical Directionality and Correlation of Low-Frequency Ambient Noise in Deep Ocean Direct-Arrival Zones

**DOI:** 10.3390/s18020319

**Published:** 2018-01-23

**Authors:** Qiulong Yang, Kunde Yang, Ran Cao, Shunli Duan

**Affiliations:** 1 School of Marine Science and Technology, Northwestern Polytechnical University, Xi’an 710072, China; yangqiulong@mail.nwpu.edu.cn (Q.Y.); npucr@mail.nwpu.edu.cn (R.C.); duanshunli@nwpu.edu.cn (S.D.); 2 Key Laboratory of Ocean Acoustics and Sensing, Northwestern Polytechnical University, Ministry of Industry and Information Technology, Xi’an 710072, China

**Keywords:** vertical directionality, correlation, low frequency, ambient noise, deep ocean

## Abstract

Wind-driven and distant shipping noise sources contribute to the total noise field in the deep ocean direct-arrival zones. Wind-driven and distant shipping noise sources may significantly and simultaneously affect the spatial characteristics of the total noise field to some extent. In this work, a ray approach and parabolic equation solution method were jointly utilized to model the low-frequency ambient noise field in a range-dependent deep ocean environment by considering their calculation accuracy and efficiency in near-field wind-driven and far-field distant shipping noise fields. The reanalysis databases of National Center of Environment Prediction (NCEP) and Volunteer Observation System (VOS) were used to model the ambient noise source intensity and distribution. Spatial vertical directionality and correlation were analyzed in three scenarios that correspond to three wind speed conditions. The noise field was dominated by distant shipping noise sources when the wind speed was less than 3 m/s, and then the spatial vertical directionality and vertical correlation of the total noise field were nearly consistent with those of distant shipping noise field. The total noise field was completely dominated by near field wind generated noise sources when the wind speed was greater than 12 m/s at 150 Hz, and then the spatial vertical correlation coefficient and directionality pattern of the total noise field was approximately consistent with that of the wind-driven noise field. The spatial characteristics of the total noise field for wind speeds between 3 m/s and 12 m/s were the weighted results of wind-driven and distant shipping noise fields. Furthermore, the spatial characteristics of low-frequency ambient noise field were compared with the classical Cron/Sherman deep water noise field coherence function. Simulation results with the described modeling method showed good agreement with the experimental measurement results based on the vertical line array deployed near the bottom in deep ocean direct-arrival zones.

## 1. Introduction

Ocean ambient noise is a kind of acoustic background, which constantly exists in the ocean and is produced by a number of different types of noise sources, including natural and man-made ones. Numerous previous studies have described the ambient noise spatial characteristics since it is a main parameter in designing sonar equipment and determining the sonar performance. Furthermore, measurements of ambient noise could also be used to infer information about the ocean acoustic environment [1,2,3,4]. 

Several theoretical expressions for wind-generated noise field had been derived in previous studies. Kuperman derived the expressions for the intensity and spatial correlation of the noise field produced in a stratified ocean by the action of wind at the surface through normal-mode representation [5]. Lee described a ray-based noise model to calculate the two-point spatial coherence function of an ocean surface generated in a hydrophone triplet array with a Maclaurin series [6]. Buckingham developed theoretical models for vertical directionality and spatial coherence based on a uniform distribution of wind-generated ambient noise surface sources in a semi-finite homogenous ocean [7,8]. Furthermore, the coherence function was derived for a sensor pair considering the effects of vertical anisotropy for three ambient noise fields of the azimuthally uniform deep ocean [9]. On the assumption that the overall directionality of the field was the product of the individual directionalities in the horizontal and vertical directions Walker conducted an analysis of the coherence and cross correlation of the noise at two sensors in 3D noise fields [10]. 

Furthermore, a large number of experiments and noise field models had been conducted to measure and simulate the spatial characteristics of noise field. The spatial coherence functions of low-frequency shipping noise derived on the basis of the Von Mises distribution showed good agreement with the experimental results in the deep ocean [11]. The spatial coherence and cross-spectral density recovered from available hydrophone pairs mounted on a deep-diving platform were consistent with a local wind-driven surface source distribution and matched that predicted by a simple model of deep water, wind-generated noise in the Tonga Trench and Philippine Sea [12,13]. Naughton presented the results where the decrease in amplitude of the noise correlation function with increased separation followed the power law on a set of free-floating oceanic receivers whose relative positions varied with time [14]. The method of frequency-wavenumber diagrams was used to examine the statistic and directional properties of ambient noise in North Pacific [15,16]. The parabolic equation solution method was applied to build the model of the vertical correlation and vertical directionality of ocean ambient noise field under the slope, seamount and varying sound speed profile environments [17]. The various approximations for noise field models based on a simple ray approach were investigated; the ray approach can produce the same accuracy as full wave treatments and can be extended to a range-dependent inhomogeneous field in the presence of non-uniform horizontal distribution of noise sources [18]. However, only one type of noise sources was considered in the theoretical derivation and simulation of spatial properties of noise field in previous researches.

In this work, the wind-generated and distant shipping noise fields may significantly and simultaneously affect the spatial characteristics of the total noise field to some extent. The spatial properties of low-frequency ambient noise field in the deep ocean direct-arrival zone were modeled and analyzed by utilizing a ray approach and parabolic equation solution method jointly. The simulation results under several wind speed conditions were consistent with experimental results measured in the South China Sea. The remainder of this paper is organized as follows: Section 2 describes the method for modeling ambient noise and theory of ambient noise spatial coherence function and directionality; Section 3 presents the numerical simulation results in the typical deep ocean for the Munk sound speed profile. Section 4 presents the experimental verification results in the South China Sea. Section 5 summarizes the discussion and conclusions.

## 2. Model and Spatial Characteristics of Ambient Noise Field

### 2.1. Methods for Modeling Ambient Noise Field

The monopole noise source is assumed to be on a plane beneath the sea surface, where the source-image pairs act as dipoles. The monopole noise sources intensity per unit area is expressed as follows:

(1) $$n_{j,l}^{2}=10^{NSL_{j,l}/10}$$

where, the monopole noise source intensity level *NSL_j,l_* depends on the location and is specified in units of decibels referenced to 1 μPa^2^/Hz at 1 m per square meter of the surface area.

**Figure 1:**
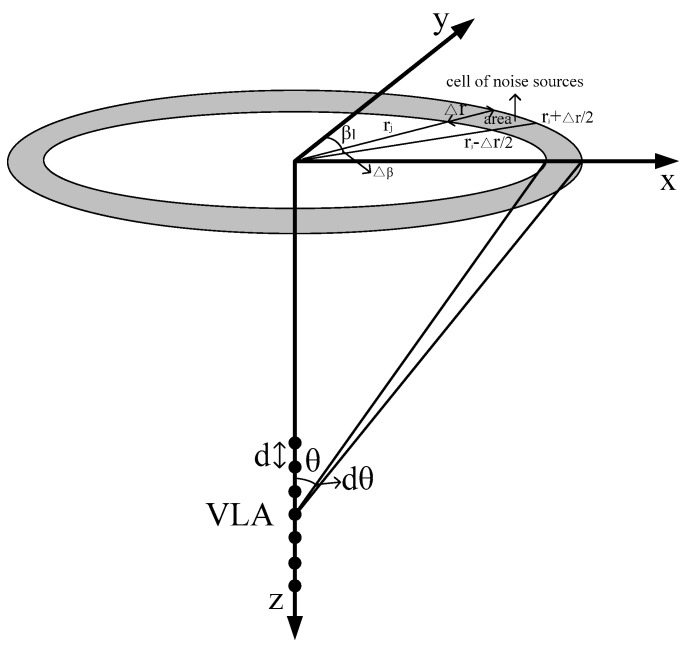
Schematic of ambient noise field modeling.

Figure 1 illustrates the schematic of modeling ambient noise field that is induced by wind agitation and distant shipping. All noise sources were scaled and combined with a random phase at a receiver position to derive the realization of the noise as follows [19,20]:

(2) $$P_{noise}\left(\bar{z}\right)=\sum_{l=1}^{L}\exp\left(i\psi_{l}\right)\sum_{j=1}^{J}\exp\left(i\psi_{j}\right)ns_{j,l}\sqrt{area_{j}}p\left(\bar{z},r_{j},z_{s},\beta_{l}\right)$$

where *ψ_j_* and *ψ_l_* are the uniformly distributed random numbers on the interval [0, 2π]; $p\left(\bar{z},r_{j},z_{s},\beta_{l}\right)$ is the complex acoustic pressure at receiver position $\bar{z}$ because of the near-surface source at range $r_{j}=r_{0}+j∆r,\;j=1,J$, depth $z_{s}$, and bearing $\beta_{l}=l∆\beta ,l=1,L$, and can be obtained through the ray approach and parabolic equation solution method for near-field wind-driven and far-field distant shipping noise fields by considering their calculation accuracy and efficiency, respectively; The time dependence exp(−*iωt*) is neglected; *ω =* 2*πf* is the angular frequency; and *f* is the frequency in Hertz.

The product of a realization of the noise and its complex conjugate at two positions $\bar{z}$ and ${\bar{z}}^{\prime }$ is expressed as follows:

(3) $$\begin{array}{cc} P_{noise}\left(\bar{z}\right)P_{noise}^{*}\left({\bar{z}}^{\prime }\right)= & \sum_{l^{\prime }=1}^{L}\sum_{j^{\prime }=1}^{J}\sum_{l=1}^{L}\sum_{j=1}^{J}\exp\left[i\left(\psi_{l}-\psi_{l^{\prime }}\right)+i\left(\psi_{j}-\psi_{j^{\prime }}\right)\right]\\ & \times ns_{j,l}ns_{j^{\prime },l^{\prime }}\sqrt{area_{j}area_{j^{\prime }}}p\left(\bar{z},r_{j},z_{s}\beta_{l}\right)p^{*}\left({\bar{z}}^{\prime },r_{j},z_{s}\beta_{l}\right) \end{array}$$

where the superscript asterisk stands for complex conjugation. The term with *l* = *l*’ and *j* = *j*’ are the same in each realization of the product. Assuming that the noise arriving from different cells (*l* ≠ *l*’ and *j* ≠ *j*’) is uncorrelated, and then the average of realizations of products of the noise is approximated as follows:

(4) $$\langle P_{noise}\left(\bar{z}\right)P_{noise}^{*}\left({\bar{z}}^{\prime }\right)\rangle ≅\sum_{l=1}^{L}\sum_{j=1}^{J}ns_{j,l}^{2}area_{j}p\left(\bar{z},r_{j},z_{s},\beta_{l}\right)p^{*}\left({\bar{z}}^{\prime },r_{j},z_{s},\beta_{l}\right)$$

where, the angular brackets represent an average over multiple realizations of the product and are called an “ensemble average”. When the noise is stationary in the statistical sense, the ensemble average can be replaced by a time average. 

The plane wave noise response can be defined briefly in terms of column vector ${\bar{p}}_{noise}=col\left[p_{noise}\left(z_{n}\right),\;n=1,\ldots ,N\right]$ which contains the complex pressure at each receiver. Then the cross-correlation or covariance matrix of complex pressure on the array can be expressed as:

(5) $$R_{noise}={\bar{p}}_{noise}{\bar{p}}_{noise}^{H}$$

where, *R_noise_* denotes the covariance matrix of noise field on the vertical line array; the superscript *H* denotes complex conjugate transpose. 

**Figure 2:**
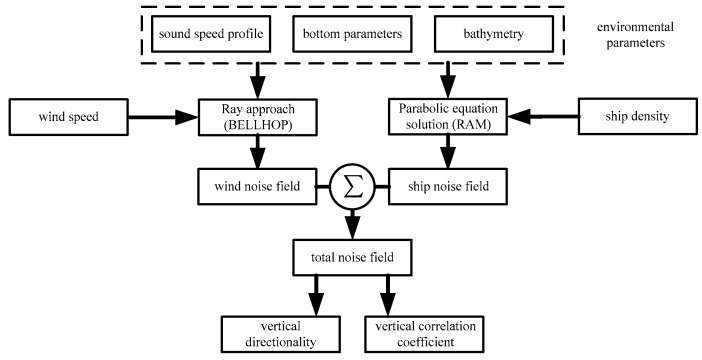
Flowchart of modeling the low-frequency ambient noise field.

Figure 2 depicts the flowchart for modeling the low-frequency ambient noise field. In addition to the environment parameters, including sound speed profiles, bottom parameters and bathymetry, wind speed and ship density were input into underwater sound propagation models based on ray approach and parabolic equation solution method for modeling noise field. The covariance matrices of the total noise field can be written as *R_total_* = *R_wind_* + *R_ship_* on the assumption that the wind noise sources and distant shipping noise sources are uncorrelated sources (where *R_wind_* and *R_ship_* represent the covariance matrices of wind-driven and distant shipping noise fields, respectively). 

### 2.2. Vertical Directionality of Ambient Noise

The coordinate positions of each element and the unit vector are $q_{n}=\left[x_{n},y_{n},z_{n}\right],\;\left(n=1,2,\ldots ,N\right)$ and $u={\left[\cos\phi \cos\theta ,\;\cos\phi \sin\theta ,\sin\phi \right]}^{T}$, respectively. Thus, the array manifold vector can be expressed as follows:

(6) $$v\left(k\right)=\left[\begin{array}{c} {\tilde{v}}_{1}\left(k\right)\exp\left(-jk^{T}q_{1}\right)\\ {\tilde{v}}_{2}\left(k\right)\exp\left(-jk^{T}q_{2}\right)\\ \vdots \\ {\tilde{v}}_{N}\left(k\right)\exp\left(-jk^{T}q_{N}\right) \end{array}\right]=\left[\begin{array}{c} v_{1}\left(k\right)\\ v_{2}\left(k\right)\\ \vdots \\ v_{N}\left(k\right) \end{array}\right]$$

where ${\left\{{\tilde{v}}_{n}\right\}}_{n=1}^{N}$ is a constant if all elements are identical; *N* is the number of array elements; *k* = −*ωu*/*c* and *τ_n_* = *k^T^p_n_*/*ω* denote the wavenumber and relative time delay, respectively; the superscript *T* denotes transposition. The beam scanning spatial power spectrum as a function of azimuth is defined as:

(7) $$P_{beam}\left(\theta ,\phi \right)=v^{H}\left(k\right)R_{noise}v\left(k\right)$$

where the superscript *H* denotes conjugate transposition; *R_noise_* represents noise data covariance matrix; *θ* and *φ* are the polar angle and azimuthal angle, respectively. 

### 2.3. Vertical Coherence of Ambient Noise Field

Two sensors were considered at positions *z*_1_ and *z*_2_ in the plane wave noise field, where the fluctuations were represented by the time series *x*_1_(*t*) and *x*_2_(*t*), respectively. The Fourier transforms of the time series were assumed as *X*_1_(*ω*) and *X*_2_(*ω*). Then, the bilateral power spectral density of the noise at the two sensors could be written as [9,13]:

(8) $$S_{jj}\left(\omega \right)=\frac{\bar{{\left|X_{j}\left(\omega \right)\right|}^{2}}}{T}$$

where, *T* is the observation time used to create the Fourier transforms; the overbar denotes an ensemble average. Similarly, the cross-spectral density of the noise fluctuations is:

(9) $$S_{12}\left(\omega \right)=\frac{\bar{X_{1}\left(\omega \right)X_{2}^{*}\left(\omega \right)}}{T}$$

where the asterisk (*) denotes complex conjugation. The coherence function Γ_12_(*ω*) is defined as the cross-spectral density that is normalized to the geometric mean of the power spectral densities at the two sensors:

(10) $$\Gamma_{12}\left(\omega \right)=\frac{S_{12}\left(\omega \right)}{\sqrt{S_{11}\left(\omega \right)S_{22}\left(\omega \right)}}$$

In general, the directional density function represents the noise power incident at the receiver in the ocean as a function of arrival angle. A convenient normalization of the directional density function, which is obtained from the total noise power integrated over all angular spaces in spherical-polar coordinates, can be written as

(11) $$\int_{0}^{2\pi }\int_{0}^{\pi }F\left(\theta ,\phi \right)\sin\theta d\theta d\phi =4\pi$$

where, *F*(*θ*,*φ*) is the directional density function and can be obtained from Equation (7). Cox derived an elegant expression which leads to an expression for the coherence function in term of directional density function, valid for arbitrary orientation of the two sensors in the plane wave noise field [21]. The expressions for coherence function are expressed as follows [9]:

(12) $$\Gamma \left(\frac{d}{\lambda }\right)=\frac{1}{2}\int_{0}^{2\pi }\int_{0}^{\pi }F\left(\theta ,\phi \right)e^{-i2\pi \frac{d}{\lambda }\cos\theta }\sin\theta d\theta d\phi \;(\mathrm{vertical})$$

(13) $$\Gamma \left(\frac{d}{\lambda }\right)=\frac{1}{2}\int_{0}^{2\pi }\int_{0}^{\pi }F\left(\theta ,\phi \right)J_{0}\left(2\pi \frac{d}{\lambda }\sin\theta \right)\sin\theta d\theta d\phi \;(\mathrm{horizontal})$$

where *J*_0_(·) is the Bessel function of the first kind of order zero; *d* and *λ* denote the distance separating sensors and the wavelength.

Cron and Sherman proposed a model of deep ocean ambient noise in which independent point sources were distributed uniformly in a horizontal plane beneath the sea surface by considering the ocean itself to be a semi-infinite, homogeneous half space. Cron and Sherman assumed straight line propagation in infinite deep water (ideal waveguide) and derived correlation coefficients for vertically and horizontally separated hydrophones The noise sources radiate sound with vertical directional density function *F*(*θ*,*ϕ*) = cos*^m^θ* (where *m* is a positive integer, usually taken as 1 or 2, and *m* = 1 corresponds to dipole sources). Then the coherence functions of the noise at the two aligned sensors are [1,9,10,13,21,22]:

(14) $$\begin{array}{cc} \Gamma \left(\frac{d}{\lambda }\right) & =2\int_{0}^{\pi /2}e^{-i2\pi \frac{d}{\lambda }\cos\theta }\cos\theta \sin\theta d\theta =2\left[\frac{-ie^{-i2\pi d/\lambda }}{2\pi d/\lambda }+\frac{e^{-i2\pi d/\lambda }-1}{{\left(2\pi d/\lambda \right)}^{2}}\right]\\ & =2\left[\frac{\sin\left(2\pi d/\lambda \right)}{2\pi d/\lambda }+\frac{\cos\left(2\pi d/\lambda \right)-1}{{\left(2\pi d/\lambda \right)}^{2}}\right]+2i\left[\frac{\cos\left(2\pi d/\lambda \right)}{2\pi d/\lambda }-\frac{\sin\left(2\pi d/\lambda \right)}{{\left(2\pi d/\lambda \right)}^{2}}\right]\;(\mathrm{vertical}) \end{array}$$

(15) $$\Gamma \left(\frac{d}{\lambda }\right)=2\int_{0}^{\pi /2}J_{0}\left(2\pi d/\lambda \sin\theta \right)\cos\theta \sin\theta d\theta =\frac{2}{2\pi d/\lambda }J_{1}\left(2\pi d/\lambda \right)\;(\mathrm{horizontal})$$

where, *J*_1_(·) is the Bessel function of the first kind of order unity.

## 3. Numerical Results of the Simulation

### 3.1. Wind Speed Dependence 

**Figure 3:**
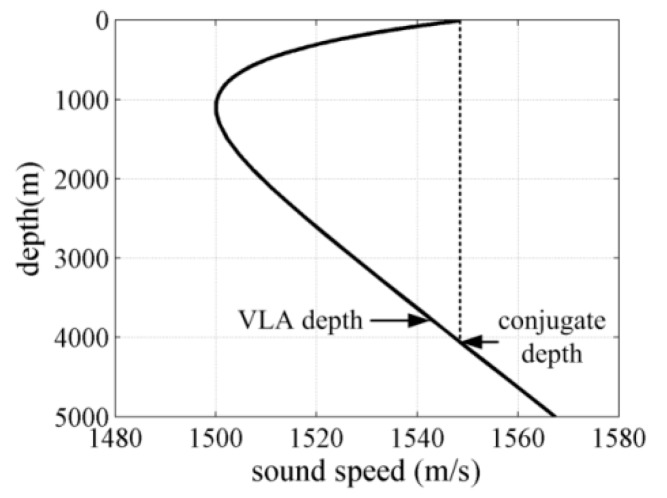
Munk sound speed profile.

Simulations were performed for the Munk sound speed profile as presented in Figure 3. The center depth of the receiver array and surface conjugate depth were assumed to be 3800 m and 4000 m, respectively. A two-layer bottom model was used to model low frequency ambient noise field. The sediment sound speed, density and attenuation were assumed to be 1550 m/s, 1.30 g/cm^3^, and 0.15 dB/λ, correspondingly. The basement sound speed, density, and attenuation were assumed to be 1650 m/s, 1.80 g/cm^3^, and 0.10 dB/λ, respectively. The thickness of the sediment was approximately 12 m. Wind agitation and distant shipping were assumed to be the dominant noise sources in the noise field. The other noise sources were disregarded in this work. The noise source depth was assumed to be on the plane of one-quarter of the wavelength beneath the sea surface for wind-driven noise [19,20,23], whereas the noise source depth was assumed to be 10 m beneath the surface for distant shipping noise. The ray approach and parabolic equation solution method could be utilized to model the wind-driven and distant shipping ambient noise fields correspondingly based on their calculation accuracy and efficiency. Therefore, the ray approach and parabolic equation solution method were jointly utilized to model the low-frequency ambient noise field on the basis of the BELLHOP [24] and RAM [25] acoustic codes. The noise source intensities of wind agitation and distant merchant ship were acquired through Research Ambient Noise Directionality (RANDI) and Ambient Noise Directionality Estimation System (ANDES) [19,23,26]. For the sake of simplicity, the distant shipping noise intensity was uncorrelated with wind speed condition in this Section. 

**Figure 4:**
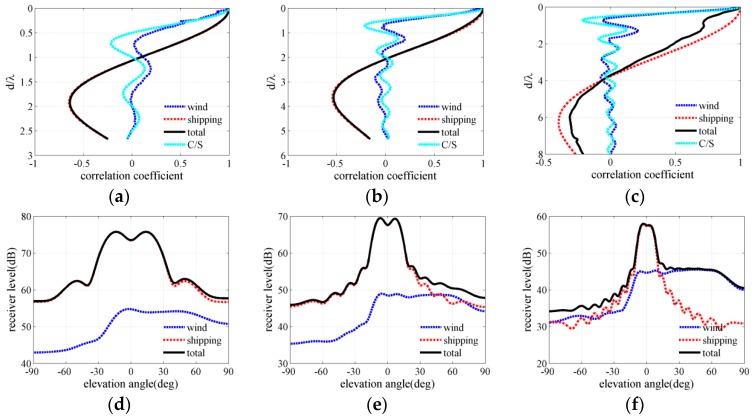
Vertical correlation and directionality at wind speed of 3 m/s for frequencies of (**a**,**d**) 50 Hz (**b**,**e**) 100 Hz and (**c**,**f**) 150 Hz.

**Figure 5:**
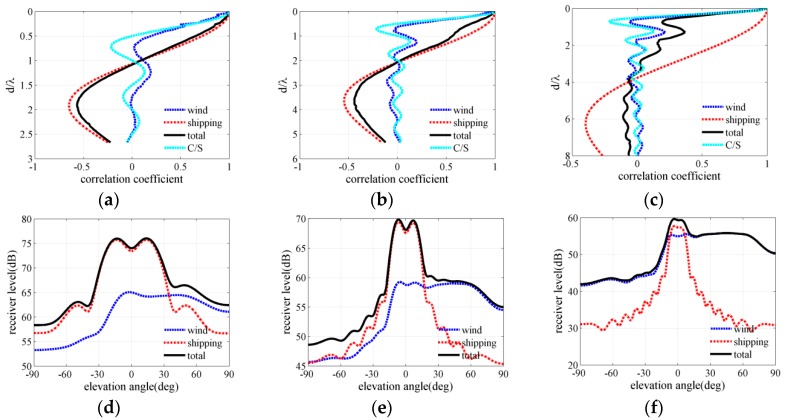
Vertical correlation and directionality at wind speed of 7 m/s for frequencies of (**a**,**d**) 50 Hz (**b**,**e**) 100 Hz and (**c**,**f**) 150 Hz.

**Figure 6:**
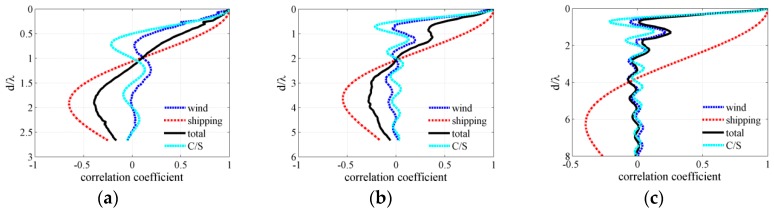

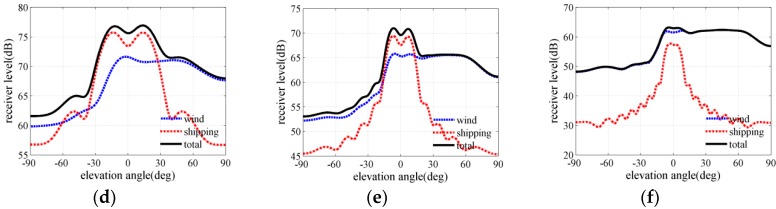
Vertical correlation and directionality at wind speed of 12 m/s for frequencies of (**a**,**d**) 50 Hz (**b**,**e**) 100 Hz and (**c**,**f**) 150 Hz.

Figure 4, Figure 5 and Figure 6 present the ambient noise vertical directionality and correlation coefficient of total noise field for wind speeds of 3, 7 and 12 m/s at 50, 100 and 150 Hz, respectively. Positive and negative angles represent the downward and upward acoustic rays arriving at the array, correspondingly. The distant shipping noise reaching the vertical line array (VLA) from shallow grazing angle was the dominant the noise source when the wind speed was 3 m/s. Dual peaks structure, which are approximately symmetrical with respect to the horizontal direction, can be observed in the vertical directionality pattern of distant shipping noise field. The directionality results were induced by upward and downward acoustic rays from the deep sound channel and along the deep sound channel or convergence zone path [27], as acoustic rays could completely reverse at the surface conjugate depth. Currently, vertical correlation and directionality result of the total noise field were consistent with those of the distant shipping noise field. The first zero radius of the shipping noise field also showed good agreement with that of the total noise field. The wind-driven noise that reached the array from relatively steep grazing angles significantly contributed to the total ambient noise field as wind speed increased. The wind-generated noise sources induced by wind agitation and breaking waves could arrive at the receiver array along the direct or reliable acoustic path [28,29,30]. Currently, the vertical correlation coefficient of the total noise field became inconsistent with that of the shipping noise field but tended toward that of the wind-driven noise field as frequency increased. The first zero radius in the vertical correlation coefficient demonstrated a complex relationship with frequency. The vertical directionality and correlation coefficient of the total noise field were completely consistent with that of the wind noise field at 150 Hz for the wind speed of 12 m/s as illustrated in Figure 6c,f. Currently, the first zero location occurs at nearly half wavelength approximately. What’s more, the vertical directionality and correlation coefficient were the weighed results of wind-driven and distant shipping noise field for wind speeds of 7 m/s. The weighted results have close relationship with the proportion of wind-driven and distant shipping noise sources in the total noise field. 

The spatial correlation coefficients in the numerical calculation results of the total noise field were compared with spatial correlation results described by Cron/Sherman (C/S). The spatial correlation coefficients of the wind-driven noise field were approximately consistent with the C/S results based on surface noise source distribution. The first zero location occurred at a half wavelength for wind-driven noise field and C/S coherence function. However, the spatial correlation coefficients of the wind noise field deviated a little from the C/S results because of the presentation of bottom reflection and sound speed gradient. Furthermore, at low wind speed condition the first zeros may have occurred at many times a half wavelength because of the presentence of distant shipping noise field. Finally, the wind-driven and distant shipping noise fields significantly and simultaneously influenced the vertical correlation coefficient and directionality pattern of the total noise field. The first zero location was determined by the competition of wind-driven and distant shipping noises in the total noise field.

### 3.2. Frequency Dependence 

**Figure 7:**
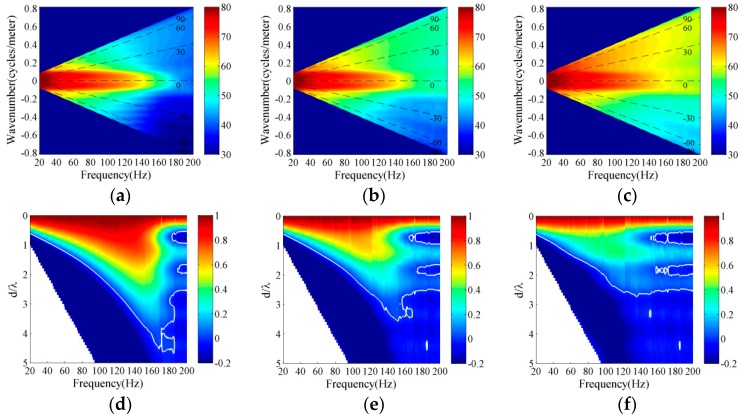
Frequency-wavenumber spectra and vertical correlation coefficient of the total noise field for wind speeds of (**a**,**d**) 3 m/s and (**b**,**e**) 7 m/s (**c**,**f**) 12 m/s.

Figure 7a–c depicts the frequency-wavenumber spectra of the total noise field for wind speeds of 3, 7 and 12 m/s. Frequency-wavenumber diagrams [15,16] were utilized to examine the vertical directionality of the ambient noise field. The dominant noise sources reaching the VLA with shallow grazing angle close to horizontal direction are originated by distant ships when the wind speed was 3 m/s. The dominant noise power originated from noise sources with relatively minimal absolute vertical wavenumber along the deep sound channel path. The near-field wind-driven noise sources may become the dominant sources when the wind speed was 12 m/s. The wind noise arrived at the array through downward acoustic rays with relatively greater positive vertical wavenumber and steep grazing angles along the direct path or reliable acoustic path. The wind-driven noise field under high wind speed condition significantly influenced the spatial vertical directionality pattern. What’s more, several diagonal lines (black dash line) were superimposed to aid the conversion of stripes with vertical wavenumber into grazing angles (±90°,±60°,±30°,0°). The general formula is given by [15]:

(16) $$\Theta =90-\cos^{-1}\left(\frac{df/dk_{z}}{c_{array}}\right)$$

where lines with a positive slope correspond to noise traveling downward the array; ±90° denotes the vertical endfire directions; *f*, *k_z_* and *c_array_* represent the frequency, vertical wavenumber and sound speed at the array, respectively.

The location of the first zeros is the critical parameter in determining the spacing separating sensors and the signal-to-noise gain of an array, and must be determined in the total noise field for surface noise source distribution. Figure 7d–f displays the vertical correlation coefficient of the total noise field for wind speeds of 3 m/s, 7 m/s and 12 m/s. The white lines in the figures represent the contour of the vertical correlation coefficient, which is equal to zero. The clear light and shade stripe in the vertical correlation coefficient diagram of frequency-depth may have been induced by near-field wind-generated noise sources. The first zero location obviously becomes short and occurs at about a half wavelength under high wind speed conditions for frequencies higher than 150 Hz. But for frequencies lower than 150 Hz, the first zero radiuses is many times a half wavelength and shows different characteristics with that in isotropic noise field where the first zero occurs at *d* = *λ*/2. Furthermore, as wind speed increase, the first zero radius of correlation coefficient becomes short and tends to a half wavelength. The first frequency at which the correlation coefficient of total noise field is equal to zeros becomes lower. At high wind speed condition the vertical correlation coefficient structure of wind generated noise field significantly influenced that of the total noise field. The first zero location at the wind speed of 3 m/s differs from that at the wind speed of 12 m/s for frequencies from 100 Hz to 160 Hz as shown in Figure 7d,f.

## 4. The Experiment Results

### 4.1. Experiment Description

**Figure 8:**
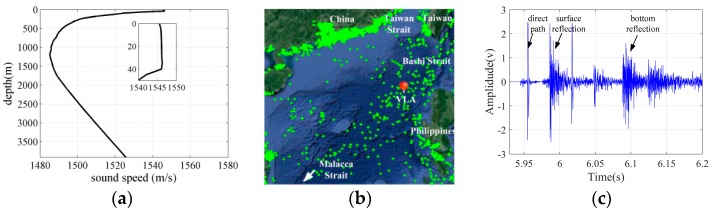
Experimental environment and signal waveform of explosive sources. (**a**) Measured sound speed profile; (**b**) real-time shipping distribution from the satellite AIS system in experiment; (**c**) received signal waveform of brand explosive sources.

An experiment was conducted in a deep ocean area of the South China Sea on 5–21 August 2016. Figure 8a demonstrates the measured sound speed profile with Conductivity-Temperature-Depth (CTD) in the experiment. The water depth and mixing layers were approximately 3910 m and 40 m, correspondingly. The acoustic waveguide is an incomplete channel due to the lack of surface conjugate depth, because the sound speed at the bottom is less than that on the sea surface. A vertical line array, which consists of 16 elements, was deployed near a main ship lane that connects Malacca and the Bashi or Taiwan Strait. Figure 8b depicts the real-time shipping distribution from the satellite Automatic Identification System (AIS) and receiver position of experiment. The green dots denote the real time position of the ships. A large number of ships traversed the VLA during the experiment measurement, and then the contribution of the shipping noise field within 50 km should be regarded as interference and should be eliminated in modeling the low-frequency noise field. The element separation was approximately 4 m, and the VLA center depth was approximately 3718 m based on depth sensors that are fixed on the VLA. The ambient noise data during the measurements were recorded continuously. The distant shipping and wind-driven noise source depths were assumed to be 10 m and a quarter of the wavelength beneath the sea surface in modeling the low-frequency ambient noise field, respectively [19,20,23]. 

**Figure 9:**
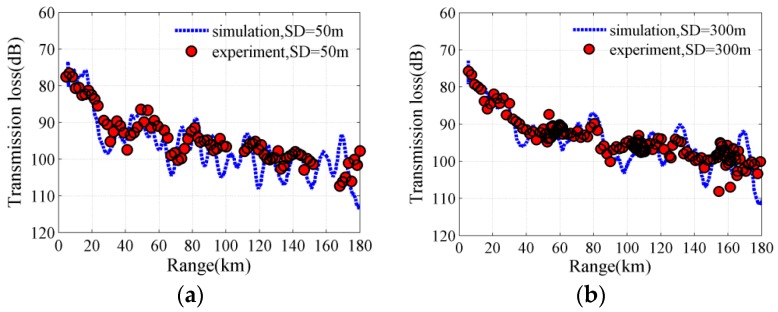
Transmission losses comparison between simulation and experiment at 100 Hz for source depths of (**a**) 50 m and (**b**) 300 m.

Furthermore, a sound propagation experiment was conducted during ambient noise recording. Broadband explosive sources were dropped from the research vessel for transmission losses measurement and geoacoustic inversion. Figure 8c depicts the received signal waveform of explosive charges for a source depth of 50 m in the experiment. The signal was received at range of 6.5 km and at receiver depth of 3715 m. The objective function, which is a function of the bottom reflection losses (BL) extracted from experimental data and calculated by underwater acoustic model, was utilized for geoacoustic parameters inversion. A two-layer bottom model was obtained from geoacoustic inversion and optimization process [31,32]. And then the two-layer bottom model was used to model the ambient noise field. Table 1 summarizes the values of the sediment and basement acoustic parameters in modeling the ambient noise field. The bottom sediment and basement acoustic parameters were inverted from experimental sound propagation data with explosive sources. Figure 9 illustrates the transmission losses comparison between simulations results using the bottom parameters listed in Table 1 and the experimental measurement results. The experimental results were measured at a receiver depth of 3715 m. The simulation results were calculated using the inverted bottom parameters based on the RAM acoustic code and were consistent with experimental measurements for source depths of 50 m and 300 m at a frequency of 100 Hz. Finally, the bottom acoustic parameters were valid and could be used to model the low-frequency ambient noise field.

### 4.2. Auxiliary Database for the Modeling Noise Field

**Figure 10:**
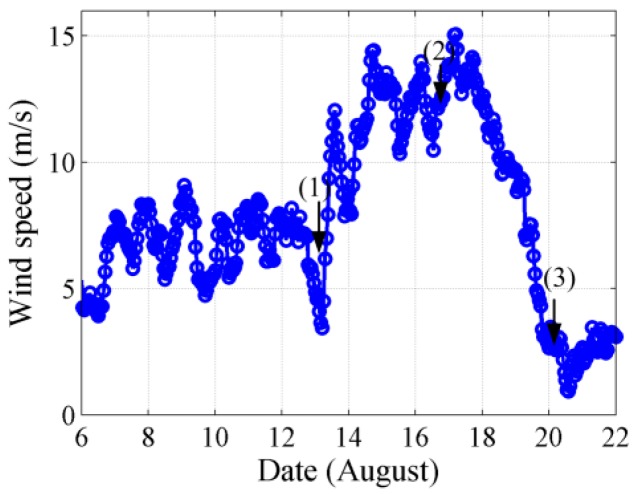
The wind speed as a function of time during experimental measurement.

**Figure 11:**
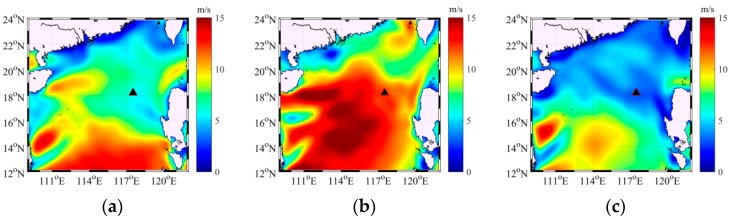
Wind speed field in experiment (unit: m/s) at (**a**) 3:00, 13 August (**b**) 22:00, 16 August (**c**) 3:00, 20 August.

Figure 10 displays the sea surface wind speed as a function of time above the VLA. The wind speed data were acquired from the National Center of Environment Prediction (NCEP) reanalysis database [33]. The wind-driven ambient noise may have significantly contributed to the total ambient noise field on 14–20 August because of the high wind speed and sea state. The numbers in brackets represent three wind speed conditions under which experimental measurement data were selected to demonstrate the effectiveness of the modeling method. The wind speed conditions at the three moments corresponded to those presented in Figure 11, which depicts the wind field at three moments in the experiment; the black triangle denotes the position of the VLA. According to the historical typhoon information of the China Meteorology Administration (CMA), the high wind speed may have been induced by typhoon “Dianmu”, which was a local cyclone in the north of the South China Sea and appeared by accident. Then, the wind field data from NCEP database was used for modeling wind-driven noise source intensity and distribution.

**Figure 12:**
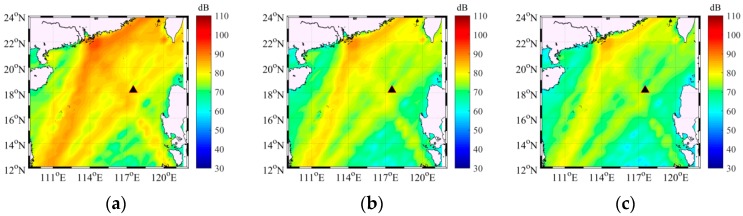
The average shipping noise source intensity based on VOS for frequency of (**a**) 50 Hz (**b**) 100 Hz and (**c**) 150 Hz (unit: dB re 1 μPa^2^/Hz/m^2^ @ 1 m).

Figure 12 demonstrates the average shipping noise source intensity based on the volunteer observation system (VOS) database for frequencies of 50, 100 and 150 Hz. VOS database, which provides information on the waypoints of global merchant ships, was used for modeling distant shipping noise source distribution. Approximately 10–12% of the global fleet provides their ship position to the VOS. The VOS ships were calculated to consist of about 82% ANDES type “merchant vessel” and 18% “large tanker” and determine the source levels (SL) of modern global fleet [34]. The global shipping data were provided in terms of kilometers of track per 10 × 10 km^2^ and then were reprocessed into the SL density maps by assuming a fixed speed to calculate the dwell or residence time for a ship in each cell. The ship count is the instantaneous number of ships instead of the number of ships that traverse the cell during a time of period. The number of new ships entering in a cell and the numbers of ships leaving the cell during a short snapshot length are assumed to be equal, thereby indicating that the average ship count in a cell remains constant. The Poisson-distribution based VOS database was used to generate 10,000 realizations, which represent 10,000 moments. For the Poisson distribution, the mean number in a cell is determined on the basis of the VOS database. Monte Carlo method based on Poisson distribution was applied to model the distant shipping noise source intensity and distribution [35]. And then the shipping noise source intensity and distribution was used to model the low-frequency ambient noise field. Furthermore, ocean bathymetry data were obtained from ETOPO1, which is a 1 arc-min global relief model developed by the National Geophysical Data Center (NGDC) [36]. The bathymetry from database was compared with aboard depthometer in the experiment and was approximately accurate in the deep ocean. The abovementioned parameters were applied to model the low-frequency ambient noise field in the experiment.

### 4.3. Experiment Verification

The acoustic field was calculated along a fan of radials with a 3° angle at each position of the receiver hydrophones in the experiments. The transmission losses were calculated along the 120 slices for frequencies of 50, 100 and 150 Hz by utilizing the standard ray approach and parabolic equation solution method jointly for low-frequency ambient noise field. The near-field wind-driven and far-field distant shipping noise fields could be calculated through the ray approach and parabolic equation solution method accurately and effectively in the range-dependent deep ocean environment, respectively. The radii along each radial were 100 and 500 km for the wind-generated and distant shipping noise fields correspondingly. The contribution of the shipping noise field within 50 km was disregarded in this work but was considered as interference in modeling the spatial properties of the noise field. The experimental measurement data at three moments, which were 3:00 13 August, 22:00 16 August and 3:00 20 August, were selected to verify the previously discussed theory considering the interference of near-field discrete ship and the wind speed above the vertical line array. At each moment, 15 min noise data that were measured with the deployed VLA were cut into several segments of 30 s data. Each segment was used to calculate the spatial vertical correlation coefficient and vertical directionality of the ambient noise field. Then, the spatial property results with all of the segments were averaged to eliminate the transient noise interference and reduce the error. The wind speeds above the VLA at three times were approximately equal to 7, 12 and 3 m/s and are illustrated in Figure 10 and Figure 11.

**Figure 13:**
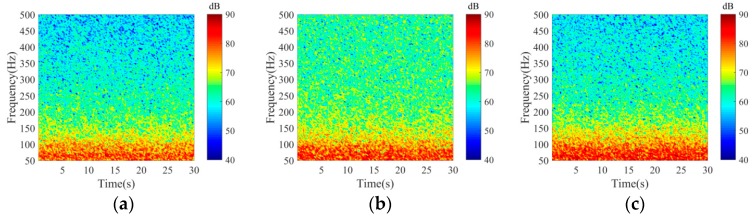
Time frequency spectrogram (unit: dB re 1 μPa^2^/Hz) in the experiment for wind speed of (**a**) 7 m/s at 3:00, 13 August, (**b**) 12 m/s at 22:00, 16 August and (**c**) 3 m/s at 3:00, 20 August.

Figure 13 shows the time frequency spectrogram in the experiment for wind speed of 3, 7 and 12 m/s which corresponded to the three moments. When the wind speed was 3 m/s, the noise field was dominated by distant shipping noise completely; when the wind speed was 12 m/s, wind driven noise had significant effects on the total noise field, received levels increased greater than 5 dB for frequencies up to 500 Hz when compared with those at wind speed of 3 m/s; when the wind speed was 7 m/s, the total noise field may be the weighted results of wind-driven and distant shipping noise field. Furthermore, the shipping density may decrease due to high sea state and high wind speed condition. Therefore, the received levels for frequency lower than 150 Hz at wind speed of 7 m/s were less than those at wind speed of 3 m/s. As the wind speed decreased, the shipping density may increase, and distant shipping noise field may have significant effects on the total noise field and take up a greater proportion in the total noise field.

**Figure 14:**
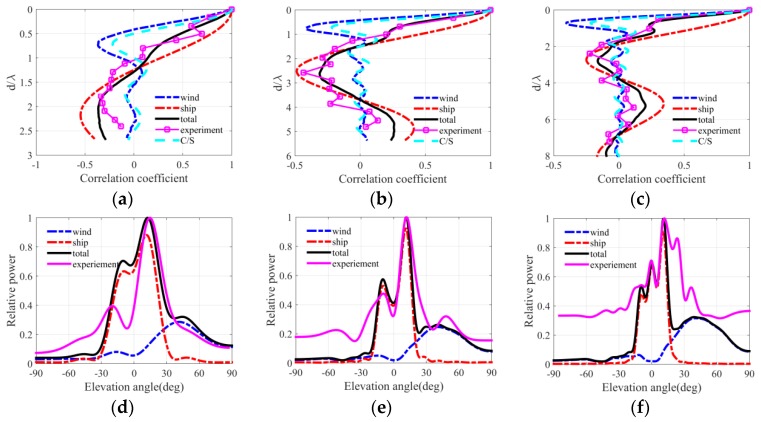
Vertical correlation coefficient and directionality at 3:00, 13 August for frequencies of (**a**,**d**) 50 Hz, (**b**,**e**) 100 Hz and (**c**,**f**) 150 Hz.

**Figure 15:**
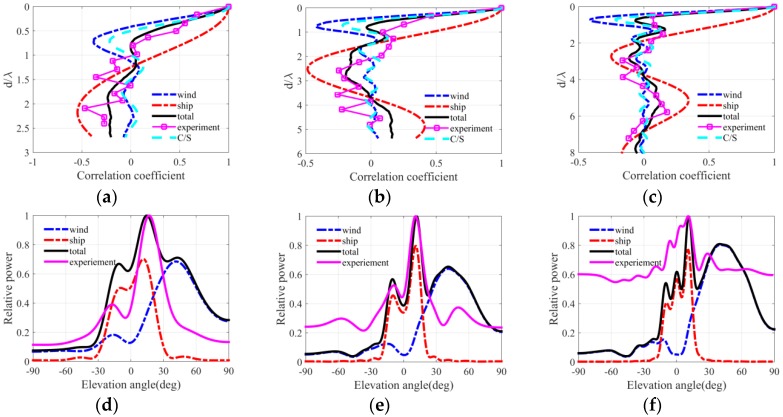
Vertical correlation coefficient and directionality at 22:00, 16 August for frequencies of (**a**,**d**) 50 Hz, (**b**,**e**) 100 Hz and (**c**,**f**) 150 Hz.

**Figure 16:**
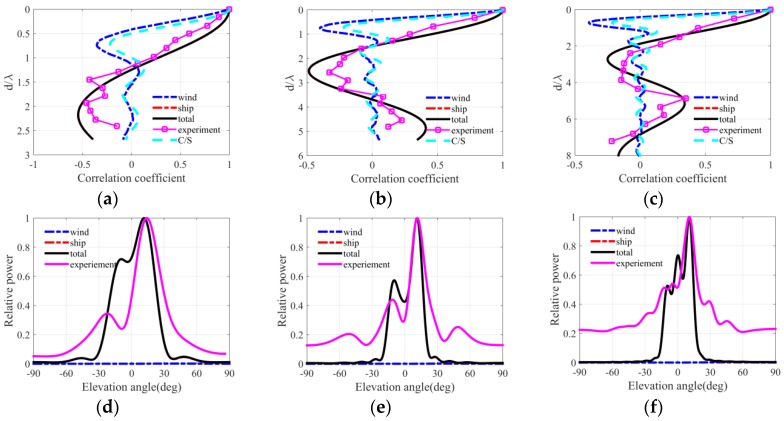
Vertical correlation coefficient and directionality at 3:00, 20 August for frequencies of (**a**,**d**) 50 Hz, (**b**,**e**) 100 Hz and (**c**,**f**) 150 Hz.

Figure 14, Figure 15 and Figure 16 depict the vertical correlation coefficient and directionality at three times that corresponded to the three wind speed conditions in the experiment. Here the received level was replaced by the normalized power for some reasons. The reference value was the maximum of original vertical directionality of total noise field. The normalized power is defined as a power relative to reference value, and the normalized power is defined as the logarithm of a ratio which is output of beam former divided by reference value in angular space. The wind-driven noise induced by wind agitation and breaking waves significantly influenced the spatial vertical correlation coefficient and vertical directionality under the high wind speed condition. In Figure 16, the distant shipping noise field was the dominant noise field when the wind speed was 3 m/s, and the spatial characteristics of the total ambient noise field were consistent with those of the shipping noise field completely. In Figure 15, the near field wind generated noise field was the dominant noise field when the wind speed was 12 m/s, and the spatial vertical directionality and correlation coefficient of the total ambient noise field were nearly consistent with those of wind driven noise field at frequency of 150 Hz. For frequencies of 50 and 100 Hz, the first zero radii were many times a half wavelength but tended to the results of the wind-driven noise field. At high wind speed condition the vertical structure of wind-generated noise field significantly influenced that of the total noise field. In Figure 14, the spatial directionality and correlation coefficient results for wind speed between 3 m/s and 12 m/s were the weighed results of the wind-generated and distant shipping noise fields. The spatial results had close relationship with the proportion of wind-driven noise and distant shipping noise source in the total noise field. Wind-driven noise and distant shipping noise arrived at the VLA through different propagation paths in the experimental oceanic environment. The wind-driven noise can reach the receiver VLA by rays along the reliable acoustic path with low transmission loss and steep grazing angle or great vertical wavenumber, whereas the distant shipping noise can reach the receiver VLA with high transmission losses and shallow grazing angle or little vertical wavenumber. The ocean acoustic channel was an incomplete channel because of the lack of surface conjugate depth. The acoustic rays could not reverse completely but were reflected by the bottom with reflection losses. Therefore, the power is less along the upward rays than along the downward rays in the noise directionality pattern of noise field. The vertical directionality of distant shipping noise exhibited an approximately symmetrical dual peak structure because of the downward and upward rays along the paths of the deep sound channel or convergence zone. 

The vertical correlation coefficient results in three wind conditions were compared with the typical Cron/Sherman deep water noise field coherence function. The vertical correlation coefficients of the C/S theory were approximately consistent with those of wind-generated noise field in numerical calculation because of the presentence of the bottom reflection and the sound speed gradient. Furthermore, the first zero location occurred at a half wavelength when the wind generated noise sources were the dominant noise source. However, the first zeros radius was multiple times a half wavelength when the total noise field was dominated by distant shipping noise sources. The simulation results of the total noise field were consistent with the experimental results of the proposed modeling method. However, several factors may induce errors in modeling the noise field spatial properties. First, the VOS database may not describe the ship distribution near the VLA completely and accurately, thereby inducing errors in the vertical directionality and correlation coefficient in the simulation results of the total noise field. Second, the noise source levels of wind and distant shipping were not accurate completely but were the empirical results. Third, the geoacoustic parameters may deviate from the inversion results far from the VLA position; besides the geoacoustic parameters, the sound speed profiles that were distant from the VLA may also be inconsistent with measured result at the position of VLA in range-dependent waveguide.

## 5. Conclusions

The array gain exhibits the enhancement of received signal-to-noise ratio (SNR) in a sonar array system. In addition, the array gain is directly related to the spatial correlation coefficient of the ambient noise field. The measurements of spatial properties of ambient noise field include considerable information on the ocean acoustic environment. Therefore, modeling the spatial correlation and directionality of the ambient noise field is necessary for sonar performance prediction and ocean environment inversion. In this work, the simulation results of the total noise field were consistent with the experimental results with the described modeling method in the direct-arrival zone in the deep ocean. Therefore, several conclusions are summarized as follows:

(1) The wind-driven and distant shipping noise field may significantly and simultaneously influence the spatial characteristics of the total noise field. The ray approach and parabolic equation solution method were jointly utilized to model the low frequency ambient noise field in the range-dependent deep ocean environment by considering their calculation accuracy and efficiency in wind-driven and distant shipping noise fields. The NCEP and VOS reanalysis databases were used to model ambient noise source intensity and distribution.
(2) The spatial vertical directionality and correlation of total noise field were analyzed in three scenarios that corresponded to three wind speed conditions. The total noise field was dominated by distant shipping noise when the wind speed was less than 3 m/s. The spatial vertical correlation and vertical directionality of the total noise field were approximately consistent with that of the shipping noise field; The near-field wind-generated noise source became the dominant noise source when the wind speed was larger than 12 m/s, and the spatial vertical correlation of the total noise field was approximately consistent with that of the wind-driven noise field at 150 Hz; Furthermore, the spatial correlation coefficient results were the weighted results of the wind-generated noise field and distant shipping noise fields for wind speeds between 3 and 12 m/s.
(3) The vertical directionality pattern of total noise field was the hybrid result of the wind-driven and distant shipping noise fields because of their different arrival paths. The wind-generated noise sources reaching the received VLA were along the direct or reliable acoustic path with low transmission loss and steep grazing angle. By contrast, the distant shipping noises arriving at the receiver VLA were through paths of deep sound channel or convergence zone with relatively high transmission loss and shallow grazing angle. The vertical directionality of the shipping noise field exhibited a symmetrical dual-peak structure along a pair of upgoing and downgoing rays along convergence zone path.
(4) The spatial correlation coefficients of the numerical results of each type of noise field were compared with those proposed by Cron/Sherman. The vertical correlation coefficient of the wind-generated noise field was nearly consistent with the C/S result. The first zero location occurred at a half wavelength when the wind generated noise sources were the dominant noise source. However, the first zeros radius was multiple times larger than a half wavelength when the total noise field was dominated by the distant shipping noise sources. The first zero location demonstrated a complex relationship with the competition of wind-driven and distant shipping noise sources in the total noise field.
(5) Several factors may induce errors in modeling the noise field spatial properties. First, the VOS database may not describe the ship distribution near the VLA completely and accurately, thereby inducing errors in the vertical directionality and correlation coefficient in the simulation results of the total noise field. Second, the noise source levels of wind and distant shipping were inaccurate but were the empirical results. Third, the geoacoustic parameters may deviate from the inversion results far from the VLA position; besides, the sound speed profiles that were distant from the VLA may be inconsistent with measured result at the position of VLA.

## Figures and Tables

**Table 1:** Sediment and basement acoustic parameters.

Bottom	Sound Speed (m/s)	Density (g/cm^3^)	Attenuation (dB/λ)	Thickness (m)
Sediment	1535–1545	1.32–1.35	0.15	0–15
basement	1560–1650	1.80	0.10	15–200
